# The theranostic performance of Chat-GPT against urological trauma

**DOI:** 10.1097/JS9.0000000000001410

**Published:** 2024-04-09

**Authors:** Jin Li, Xianyanling Yi, Zeyu Han, Dazhou Liao, Tianyi Zhang, Liangren Liu, Jianzhong Ai

**Affiliations:** a Department of Urology, Institute of Urology, West China Hospital; b West China School of Medicine, Sichuan University, Chengdu, China

*Dear Editor*,

Developed by OpenAI, Chat-GPT (Generative Pre-trained Transformer) is a powerful language model capable of dealing with raised problems with a new level of interactivity, speed, and usability. In a recent article published in the International Journal of Surgery elaborating on the application of Chat-GPT in surgical diagnosis and treatment, the results displayed that Chat-GPT is potentially good, yet faces several limitations^1^. However, another study^2^ showed that Chat-GPT is not a qualified neurosurgeon when encountering neurosurgical medicine practice, which indicated that Chat-GPT might implicate distinctly in different realms of surgical subspecialties. In this study, we delve into investigating the performance of Chat-GPT in the diagnosis and treatment of urological trauma (including renal trauma, ureteral trauma, bladder trauma, urethral trauma, and genital trauma), which could test the potential ability and application of Chat-GPT in urological trauma management.

We selected 56 questions about urological trauma from clinical recommendations in three guidelines, including the American Urological Association (AUA)^3^, the European Association of Urology (EAU)^4^ and World Society of Emergency Surgery and the American Association for the Surgery of Trauma (WSES-AAST)^5^. And thereafter, we conducted inquiries with Chat-GPT (https://chat.openai.com/) to collect responses (Accessed on 27 Feb, Supplementary Table 1.). Next, each response was assessed by three independent urological experts according to the aforementioned guidelines and their own clinical experience. More specifically, the accuracy of responses was graded according to the five-point Likert score system: 1 means completely incorrect; 2 means more incorrect than correct; 3 means equally incorrect and correct; 4 means more correct than incorrect; 5 means completely correct. Furthermore, the median score from three experts was determined as the final score in a bid to decrease the statistical bias.

Generally, Chat-GPT would provide comprehensive diagnostic criteria and principles of treatment when posed with theragnostic questions associated with urological trauma. According to the results (Tables 1), 1 (1.79%) response was rated as 1, 3 responses (5.36%) were rated as 2, and 6 (10.71%), 8 (14.26%) and 38 (67.86%) were rated as 3, 4 and 5, respectively. Roughly 82.14% of responses had reached mostly or completely correct. However, the only response that got 1 score comes from “When feasible, is early endoscopic re-alignment strongly recommended in male pelvic fracture urethral injuries (PFUIs)?”. The guidelines from EAU weakly recommend this management due to contradictory evidence about the prognosis in male PFUIs who have received early endoscopic re-alignment. And it’s interesting to note that the discrepancies between the three urological experts on those low-score responses are small, indicating that these responses generated by Chat-GPT were thoroughly not relevant to the guideline recommendations and clinical medicine practice.

**Table 1:** Five-point Likert scores for responses from inquires posed to Chat-GPT.

	Five-point Likert scores^a^			
Questions	Expert1	Expert2	Expert3	Median
1. What should the doctors record in patients upon admission when there is suspicion of renal injury?	4	4	5	4
2. Is extended-focused abdominal sonography effective and rapid to detect intra-abdominal free fluid?	5	5	5	5
3. Does abdominal sonography have high sensitivity and specificity in kidney trauma?	4	4	4	4
4. What should be performed in trauma patients with visible haematuria or microscopic haematuria and one episode of hypotension or penetrating trauma or clinical signs suggesting renal trauma?	5	5	5	5
5. Are operations required for stable patients with blunt, mild stab or mild gunshot renal trauma?	4	5	5	5
6. What should be performed for active renal bleeding if there are no other indications for immediate surgical exploration?	5	5	5	5
7. What should be performed in patients with kidney trauma when there are persistent or symptomatic urinary leak?	5	5	5	5
8. Is US, contrast-enhanced US, and eco-doppler recommended for pregnant women and in the paediatric population with hemodynamically stable kidney trauma?	5	5	5	5
9. Should surgeons start renal exploration in the presence of persistent hemodynamic instability, severe penetrating injury or pulsatile peri-renal haematoma?	5	5	5	5
10. Is renal reconstruction recommended in patients with kidney trauma when haemorrhage is controlled and there is sufficient viable renal parenchyma?	3	2	2	2
11. Is repeat imaging required in penetrating kidney injuries or in cases of fever, worsening flank pain, or falling haematocrit?	5	5	5	5
12. Are urinalysis, radiological investigation and blood pressure measurement recommended for the follow up of major renal injury?	5	5	5	5
13. Is isolated urinary extravasation an absolute contra-indication to NOM in absence of other indications for laparotomy?	5	4	5	5
14. What is indicated in patients with severe main renal vein injury without self-limiting bleeding?	5	5	5	5
15. Is visually identifying the ureters to prevent ureteral trauma recommended during complex abdominal and pelvic surgery?	5	5	5	5
16. Which kind of injury should be aware of in all abdominal penetrating trauma, and in deceleration-type blunt trauma?	5	5	5	5
17. Are pre-operative prophylactic stents recommended in high-risk cases?	5	5	5	5
18. Should iatrogenic ureteral injuries recognized during surgery be repaired immediately?	5	5	5	5
19. What should be used in iatrogenic ureteral injuries with delayed diagnosis?	5	5	5	5
20. What kind of imaging study should be performed in stable trauma patients with suspected ureteral injuries?	4	4	4	4
21. When possible, what should clinicians initially manage patients with ureterovaginal fistula?	5	5	5	5
22. What kinds of management is recommended in endoscopic ureteral injuries when possible?	5	5	5	5
23. What kind of management is required when urine flow is impaired in patients with ureter contusions?	4	4	4	4
24. In kidney trauma patients not suitable for NOM, what should be performed in partial and complete ureteral transections or avulsion in cases in distal lesions?	5	5	5	5
25. If placing ureteral stenting fails, what is indicated in cases of partial ureteral injuries diagnosed in a delayed fashion?	4	4	4	4
26. Is cystography or CT recommended in the presence of visible haematuria, pelvic fracture or suspected iatrogenic bladder injury in the post-operative setting?	5	5	5	5
27. What should be performed to rule out bladder injury during retropubic sub-urethral sling procedures?	5	5	5	5
28. Is NOM recommended in uncomplicated blunt extraperitoneal bladder injuries?	3	2	2	2
29. Are surgical interventions recommended in blunt extraperitoneal bladder injuries in cases of bladder neck injuries?	5	5	5	5
30. What should be performed in blunt or penetrating intraperitoneal injuries?	5	5	5	5
31. Is NOM recommended in small uncomplicated intraperitoneal bladder injuries during endoscopic procedures?	2	3	3	3
32. What should be performed to assess bladder wall healing after repair of a complex injury or in case of risk factors for wound healing?	3	3	2	3
33. What should be performed in patients following surgical repair of bladder injuries without suprapubic cystostomy?	5	5	5	5
34. Are methylene blue or indigo carmine useful in intraoperative investigation of intraperitoneal bladder injuries?	5	5	5	5
35. Is bladder contusion treated conservatively appropriate?	5	5	5	5
36. What kind of diagnostic method is recommended to evaluate male urethral injuries?	3	3	3	3
37. What kind of diagnostic method is recommended to evaluate female urethral injuries?	4	4	3	4
38. To achieve urinary diversion, which pathway is recommended to treat iatrogenic anterior urethral injuries?	3	3	2	3
39. Is suprapubic or urethral catheterization recommended to treat partial blunt anterior urethral injuries?	4	4	3	4
40. If surgical expertise is available, is immediate urethroplasty strongly recommended to treat complete blunt anterior urethral injuries?	5	5	5	5
41. When feasible, is early endoscopic re-alignment recommended in male PFUIs?	1	1	2	1
42. Are repeat endoscopic treatments required after failed re-alignment for male PFUIs?	2	2	1	2
43. Is suprapubic or transurethral catheter recommended to initially treat partial posterior urethral injuries?	3	4	3	3
44. Is immediate urethroplasty (< 48 h) initially recommended in male PFUIs?	5	5	5	5
45. Is early urethroplasty (2 days–6 weeks) recommended for some selected male PFUIs with complete disruption?	5	5	5	5
46. What is recommended to treat complete posterior urethral disruption in male PFUIs?	4	3	3	3
47. Is early repair (within 7 days) recommended for female PFUIs?	5	5	5	5
48. What should be monitored in patients for following urethral injury for at least one year?	5	5	5	5
49. Is conservative treatment generally recommended for penetrating urethral injuries?	5	5	5	5
50. Which diagnostic method is recommended to diagnose testis trauma?	5	5	5	5
51. What is recommended to treat penile fractures?	5	5	5	5
52. What is recommended to treat testicular rupture and most penetrating scrotal injuries?	5	5	5	5
53. What should be suspected when a patient presents with penile ecchymosis, swelling, pain cracking or snapping sound during intercourse and immediate detumescence?	5	5	5	5
54. Which diagnostic method is recommended to diagnose patients with equivocal signs and symptoms of penile fracture?	5	5	5	5
55. What should be performed in patients with extensive genital skin loss or injury from infection, shearing injuries, or burns?	4	5	4	4
56. What should be performed in patients with traumatic penile amputation, with the amputated appendage wrapped in saline-soaked gauze, in a plastic bag and placed on ice during transport?	5	5	5	5

CT, computed tomography; NOM, non-operative management; PFUI, pelvic fracture urethral injury; US, ultrasound.

a Five-point Likert score system: 1 means completely incorrect; 2 means more incorrect than correct; 3 means equally incorrect and correct; 4 means more correct than incorrect; 5 means completely correct.

To summarize, the performance of Chat-GPT is quite qualified and reliable in terms of theranostic in urological trauma. It holds immense potential to generate an accurate and comprehensive response with high probabilities posed by the operators. The accuracy of Chat-GPT rests on the training datasets and the quality of the inquiries. And it appears that the more restricted and detailed the question is, the more accurate and precise the answer Chat-GPT could provide. However, there are some concerns about its extensive utilization in the field of urological trauma. Chat-GPT could provide rough general principles while even every response ended up with an inquiry with urological specialists and collaboration with the multidisciplinary team. Additionally, in consideration of its specialty, problems related to security, ethics and legality upon the utilization of Chat-GPT also need to be addressed. Standardized supervision departments and perfect law and regulation systems targeted at Chat-GPT are also warranted. So far, the application of Chat-GPT could only serve as an auxiliary tool, which requires urological doctors to sift the wheat from the chaff from the responses generated by Chat-GPT to reach the optimal choice for each patient based on their characteristics.

## Ethical approval

Not applicable.

## Consent

None.

## Sources of funding

None.

## Author contribution

J.Z.A., J.L., X.Y.L.Y. and Z.Y.H.: conceptualization, writing—original draft; D.Z.L. and T.Y.Z.: data curation; J.Z.A. and L.R.L.: writing—review and editing and supervision. All authors contributed to and revised the submitted version of the paper.

## Conflicts of interest disclosure

The authors declare no conflicts of interest.

## Research registration unique identifying number (UIN)

None.

## Guarantor

None.

## Data availability statement

All data are included in the manuscript.

## Provenance and peer review

None.
